# Dietary Diversity and Its Contribution to the Magnitude of Anaemia among Pregnant Women: Evidence from Rural Areas of Western China

**DOI:** 10.3390/nu15173714

**Published:** 2023-08-25

**Authors:** Zhengjie Cai, Linhua Li, Jieyuan Feng, Hein Raat, Yuju Wu, Huan Zhou, Scott Rozelle

**Affiliations:** 1 Department of Health Behavior and Social Medicine, West China School of Public Health and West China Fourth Hospital, Sichuan University, No. 16 South Renmin Road 3 Section, Chengdu 610041, China; caizhengjie@stu.scu.edu.cn (Z.C.); lilinhua_9@163.com (L.L.); 2 Freeman Spogli Institute for International Studies, Stanford University, Stanford, CA 94305, USA; cindyfn@stanford.edu (J.F.); rozelle@stanford.edu (S.R.); 3 Department of Public Health, Erasmus University Medical Center, 3000 CA Rotterdam, The Netherlands

**Keywords:** anaemia, dietary diversity, pregnant women, rural China

## Abstract

Background: Prenatal anaemia causes serious consequences for both mother and foetus, and dietary factors are suggested to be associated with anaemia. However, research in pregnant women living in rural areas is limited. We aim to assess the contribution of dietary diversity to the magnitude of prenatal anaemia in rural China and identify the interactions between dietary diversity and several sociodemographic and maternal characteristics in relation to anaemia. Methods: A multi-stage random cluster sampling method was used to select pregnant women in rural western China. The Woman’s Dietary Diversity Score was created to measure dietary diversity, which was recoded into terciles. Multinomial logistic regression models were used to assess the associations between dietary diversity score terciles and the magnitude of prenatal anaemia. Multiplicative interactions were tested by adding the product term of dietary diversity and several sociodemographic and maternal characteristics into the regression models. Results: Out of 969 participants, 54.3% were measured as anaemic, with 28.6% mildly anaemic and 25.7% moderately to severely anaemic. There was an absence of agreement between self-reported and measured anaemia status (κ = 0.28, 95% CI [0.22–0.34]). Participants in the highest dietary diversity score tercile had lower odds of being moderately to severely anaemic after adjusting for potential confounders (RRR = 0.65, 95% CI [0.44, 0.98]). In participants with moderate to severe anaemia, significant interactions were found between dietary diversity score terciles, age, and parity (*p* for interaction < 0.05). Conclusions: The prevalence of prenatal anaemia in rural China remains high, and pregnant women living in these areas are insufficiently aware of their anaemia status. Improving dietary diversity is needed to manage prenatal anaemia in rural areas.

## 1. Introduction

Anaemia, a condition where the number of red blood cells in the body is lower than normal, remains a critical global public health concern [1]. According to estimates by the World Health Organization (WHO), 38% of pregnant women aged 15–49 years worldwide are anaemic, with prevalence rates especially high in areas with low development [2]. Evidence shows that pregnant women are one of the most vulnerable groups to anaemia due to their increased micronutrient requirements and the physiological changes they experience during the course of their pregnancy [3]. In addition, anaemia during pregnancy (or prenatal anaemia) has been associated with numerous short- and long-term negative consequences on the health outcomes of both mothers and their children [4,5]. Promoting healthy pregnancy is thus essential for ensuring health over the life course and enhancing social and economic well-being.

In China, despite substantial improvements in maternal and child health over the past three decades, reducing the nationwide prevalence of prenatal anaemia and narrowing regional differences in prevalence remain challenges [6]. For example, according to the latest data published by the Chinese Centre for Disease Control and Prevention, the overall prevalence of prenatal anaemia was found to be 41.98% among 206,753 pregnant women [7]. Additionally, a meta-analysis conducted in 2018 found that the prevalence of prenatal anaemia in China ranged from 4.8% to 51.4%, and that out of China’s eastern, central, and western regions, prenatal anaemia rates were highest in its western regions [8]. As evidenced by these reports, China remains far from achieving the targets set by the National Nutrition Program (2017–2030) and the Healthy China Action Plan (2019–2030) aimed at reducing China’s nationwide prenatal anaemia prevalence to 10% by 2030 [9,10]. Hence, research identifying the modifiable risk factors behind the high rates of prenatal anaemia in China should be strongly prioritized in order to guide the development of strategies for preventing and managing prenatal anaemia. In particular, it is crucial to promote appropriate strategies tailored for socioeconomically disadvantaged regions [6].

Improving diet quality has been identified as one of the most important and cost-effective strategies for preventing and managing prenatal anaemia [11]. Poor nutrition is one of the most common causes of anaemia; in particular, a deficiency in key nutrients such as iron, folate, vitamin B-12, and vitamin A can lead to the development of nutritional anaemia [12]. As a woman’s requirements for micronutrients, such as iron, increase during pregnancy, consuming a diverse diet is essential to ensure adequate intake of micronutrients [13]. Maintaining optimal dietary intake and dietary diversity throughout pregnancy, however, remains a challenge for women living in less developed areas [14]. Evidence has shown that pregnant women living in less developed areas tend to have cereal- and plant-based diets, under-consuming animal-based foods, fruits, and vegetables [14,15]. Another study conducted in a rural area of western China found that 97%, 91%, and 64% of the pregnant women surveyed had inadequate amounts of folate, zinc, and iron intake, respectively [16]. Moreover, in 2020, Ma et al. investigated the maternal health behaviour of pregnant women in rural China and reported that 61% of the pregnant women surveyed had low dietary diversity [17]. According to this literature, addressing the poor dietary intake and dietary diversity of pregnant women is essential for reducing the burden of prenatal anaemia in rural China.

Despite the findings described above, there remain gaps in the existing literature. Previous studies investigating the linkage between dietary factors and prenatal anaemia mainly examine the associations between a single food group or micronutrient and prenatal anaemia [18,19,20]. Foods, however, are not consumed in isolation; it is necessary to consider how overall diet quality impacts anaemia, rather than the impact of a certain food group. In light of this, the Food and Agriculture Organization of the United Nations has proposed a measure known as dietary diversity for evaluating overall diet quality [21]. Compared with other diet quality instruments, dietary diversity is simpler, more cost-effective, and convenient, and has been designed to be user-friendly [22]. Additionally, dietary diversity has been validated among women aged 15–49 years; Arimond et al. found that dietary diversity scores were strongly correlated with the micronutrient adequacy of the diet for this population [23]. Finally, to the best of our knowledge, few studies have examined the associations between dietary diversity and prenatal anaemia in the context of rural China.

Thus, we have three objectives. First, we describe the prevalence of prenatal anaemia and the dietary intake of pregnant women living in the rural areas of western China. Importantly, we note that because of our hypothesis that pregnant women living in rural China may not be aware of their anaemia status (either due to not exhibiting obvious clinical symptoms of anaemia or because they are likely to receive substandard prenatal care), we calculate the agreement between self-reported anaemia status given by the participants and measured anaemia status tested by the research team. Second, we explore the associations between dietary diversity and the magnitude of prenatal anaemia. Another important question that has not been answered concerning the impact of dietary diversity on prenatal anaemia lies in whether additional sociodemographic and maternal characteristics are a modifier variable in relation to dietary diversity. Thus, finally, we assess the interactions between sociodemographic and maternal characteristics and dietary diversity in relation to the magnitude of prenatal anaemia.

## 2. Materials and Methods

### 2.1. Study Design and Participants

This study is part of a larger cohort study on maternal and child health carried out in China’s rural regions. We use data from three waves of repeated cross-sectional surveys (Wave 1: November to December 2019; Wave 2: July to August 2021; Wave 3: April to May 2022), all of which were conducted in the same rural areas of western China. Pregnant women were included in each wave of data collection. This study was approved by the Institutional Review Boards at Sichuan University (protocol K2019046) and Stanford University (protocol 44312). All study participants provided their informed consent prior to enrolment and understood that their participation was purely voluntary.

#### Sampling

Participants for this study were enrolled according to a three-step multi-stage random cluster sampling method. First, we randomly selected four counties from one prefecture in Sichuan Province. Second, we randomly selected 20 townships from each county. Third, we recruited all pregnant women in each sampled township according to a list of pregnant women provided by the township’s local offices of maternal and child health. Taking into account the decreasing fertility rate and the growing population of rural-to-urban migrants during recent years in China [24], we randomly selected 10 more townships from each county in the second and third survey waves.

We included all pregnant women in their second and third trimesters of pregnancy as participants. We excluded participants with chronic health conditions or major diseases (i.e., tuberculosis, HIV/AIDS, or serious mental trauma); participants who refused to give consent to measure their haemoglobin levels also were excluded. After following the above protocols, our final sample included 969 participants from 119 rural townships in western China.

### 2.2. Data Collection

The research team collected three rounds of data and all the data collection processes were overseen by trained field supervisors. During each survey round, trained survey enumerators collected data using a structured, in-person questionnaire conducted through home visits. For each participant, the survey teams collected three blocks of data: (a) information on dietary diversity; (b) sociodemographic characteristics; and (c) maternal characteristics.

#### 2.2.1. Dietary Diversity

We assessed dietary diversity with a questionnaire asking if participants had consumed food items belonging to 14 different food categories in the 24 h prior to survey conduction. The measurement method for dietary diversity is based on recommendations from the Food and Agriculture Organization (FAO) of the United Nations [21]. Using one 24 h recall period provides an assessment of the diet at the population level and is useful for monitoring progress or performing interventions [25]. Moreover, compared with longer timeframes for recall, a 24 h period was chosen as it may be less subject to recall error and less cumbersome for a respondent, and also conforms to the recall time period used in many dietary diversity studies [23,25,26,27]. Then, based off their answers to the questionnaire, we computed a Women’s Dietary Diversity Score (WDDS) for each participant. To calculate the WDDS, we classified each food item a participant reported consuming in the previous 24 h before survey conduction into one of nine different food groups: (1) starchy staples; (2) dark green leafy vegetables (DGLVs); (3) other vitamin A-rich fruits and vegetables; (4) other fruits and vegetables; (5) organ meat; (6) meat and fish; (7) eggs; (8) legumes, nuts, and seeds; and (9) milk and milk products. If a participant reported consuming at least one food item within a food group, consumption for that food group was recorded as ‘yes’; otherwise, it was recorded as ‘no’. To arrive at a participant’s final WDDS, each food group marked as consumed (i.e., had been recorded as ‘yes’) added 1 point to their WDDS, with the maximum score being 9. Finally, each participant’s WDDS was recoded into a tercile (low, medium, or high) in order for the dose–response relationship with anaemia to be analysed.

#### 2.2.2. Sociodemographic and Maternal Characteristics

The survey enumerators also collected data on sociodemographic and maternal characteristics from each participant that included the following: (a) the participant’s age (16–24 years, 25–29 years, or 30–45 years); (b) the participant’s education level (primary school or below, junior high school, senior high school, college or above); (c) if the participant had a primary occupation (yes or no); (d) the size of the participant’s immediate family (≤4 or ≥5); (e) the participant’s gestational weeks of pregnancy (second trimester or third trimester); (f) participant parity (primiparous or multiparous); (g) whether any iron or folic acid supplements had been taken by the participant during the course of their pregnancy (yes or no); and (h) if the participant self-reported having anaemia during their pregnancy prior to the research team collecting haemoglobin samples (yes or no). Additionally, we calculated household wealth indexes for each participant’s household using principal component analysis [28]. Depending on their household wealth index scores, participants were grouped into terciles of socioeconomic status (SES) that classified their SES as either low, medium, or high.

#### 2.2.3. Haemoglobin Concentrations

Haemoglobin (Hb) concentrations were collected for each participant to determine whether they had anaemia and, if so, the magnitude of the anaemia. Capillary blood samples were collected from each participant by trained team members with a HemoCue Hb 201+ fingertip prick system (Hemocue, Inc., Ängelholm, Sweden) [29]. Following the WHO’s cut-off points of prenatal anaemia, we categorized the participants’ anaemia status as ‘no anaemia’ (Hb levels of 110 g/L or greater), ‘mild anaemia’ (Hb levels of 100–109 g/L), or ‘moderate to severe anaemia’ (Hb levels of less than 100 g/L). We also collected information on the altitudes of the research areas. The participants in this study resided at altitudes below 1000 m above sea level, so no adjustments to these cut-off values are necessary. The Hb levels and the anaemia status of the participants were the primary outcomes of this study.

### 2.3. Statistical Analysis

In this study, the statistical analysis is comprised of four parts. First, descriptive analyses were carried out using frequencies and percentages for categorical variables and means and standard deviations (SD) for continuous variables. Missing values were imputed using regression imputation. We categorized raw WDDSs into terciles since we believed that would enable us to better discriminate between participants with low-quality diets and participants with higher-quality diets and provide us with an opportunity to examine dose response.

Second, chi-square tests were used to assess differences in sociodemographic and maternal characteristics by categories of anaemia as well as to assess differences in single food consumption by WDDS terciles. Cohen’s kappa statistics were computed to determine the degree of agreement between measured and self-reported anaemia status categories.

Third, three multinomial logistic regression models were designed to assess the associations between the WDDS terciles and the magnitude of anaemia. During this part of the analysis, due to the low proportion of participants with severe anaemia in our sample, anaemia status was divided into three categories instead of four: (a) no anaemia; (b) mild anaemia; and (c) moderate to severe anaemia. Unadjusted models were used to assess the associations between the WDDS terciles and the categories of anaemia status. Model 1 was adjusted for survey year, maternal age, occupation, education, SES, family size, gestational age, and parity; Model 2 was further adjusted for the level of iron supplement intake and folic acid supplement intake during pregnancy. Relative risk ratios (RRR) and their 95% confidence intervals (CIs) were calculated in the models. As we also analysed the effects of the WDDS terciles on Hb levels through multiple linear regression analyses, the adjusted covariates in Model 3 and Model 4 are the same as Model 1 and Model 2, respectively. Finally, the multiplicative interaction terms between the WDDS and maternal age, parity, gestational age, and iron and folic acid supplement intake during pregnancy were tested by adding the product of the variables into the regression models.

In all cases, we used a cluster-corrected estimator to adjust standard errors for clustering at the township level. All analyses were performed using STATA software (version 16.0; Stata Corporation, College Station, TX, USA). The results were considered statistically significant when *p* values were below 0.05. 

## 3. Results

### 3.1. Descriptive Statistics

Table 1 shows the sociodemographic and maternal characteristics of the participants. A total of 969 pregnant women were sampled, with the 25–29-year-old age group accounting for the largest proportion (40.4%). Less than half (40.4%) of all participants had a senior high school degree or above, and the majority (79.2%) of participants did not have a primary occupation (that is, they were primarily living at home without a full- or part-time job at the time of the survey). The family size of slightly over half (53.3%) of the participants was four or less. Regarding maternal characteristics, 43.9% of the sample were in their second trimester of pregnancy and 56.1% of the sample were in their third trimester of pregnancy. The majority (65.8%) of the participants were multiparous (i.e., had already given birth to one or more children). Finally, 89.1% of participants reported using folic acid supplements during pregnancy and 39.5% of participants reported using iron supplements during pregnancy.

### 3.2. Anaemia Status: Measured Versus Self-Reported Rates

**Figure 1:**
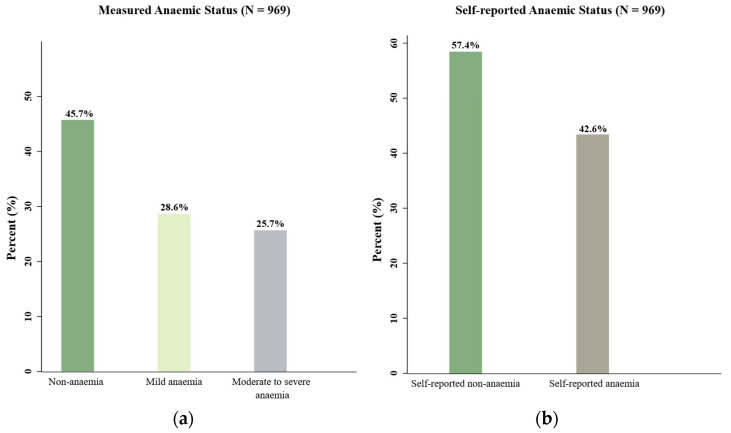

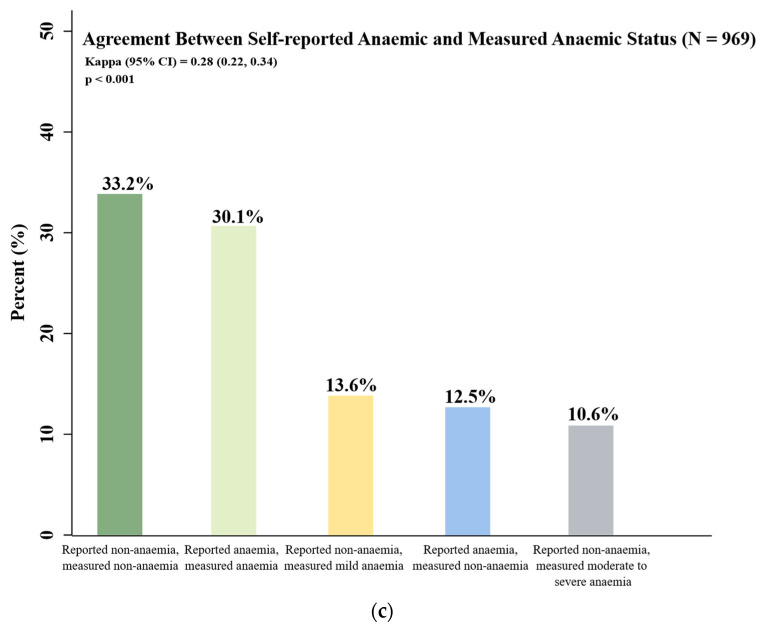
Prevalence of measured anaemia and self-reported anaemia and the agreement between measured anaemia and self-reported anaemia status among pregnant women in rural Sichuan Province in China (N = 969).

Figure 1a shows the overall prevalence of measured anaemia in the participants to be 54.3%, with 28.6% of participants found to be mildly anaemic and 25.7% of participants found to be moderately to severely anaemic. When asked to self-report their anaemia status prior to the finger-prick test, 42.6% of participants had reported being anaemic (Figure 1b). Importantly, as shown by the results in Figure 1c, there was indeed an absence of agreement between self-reported and measured anaemia status (κ = 0.28, 95% CI [0.22–0.34], *p* < 0.001). Although 57.4% of all participants had self-reported that they were not anaemic, 24.2% of these participants were then measured to be anaemic. Of the 24.2% measured to be anaemic, 10.6% were found to be moderately to severely anaemic and 13.6% mildly anaemic.

### 3.3. Dietary Diversity

**Figure 2:**
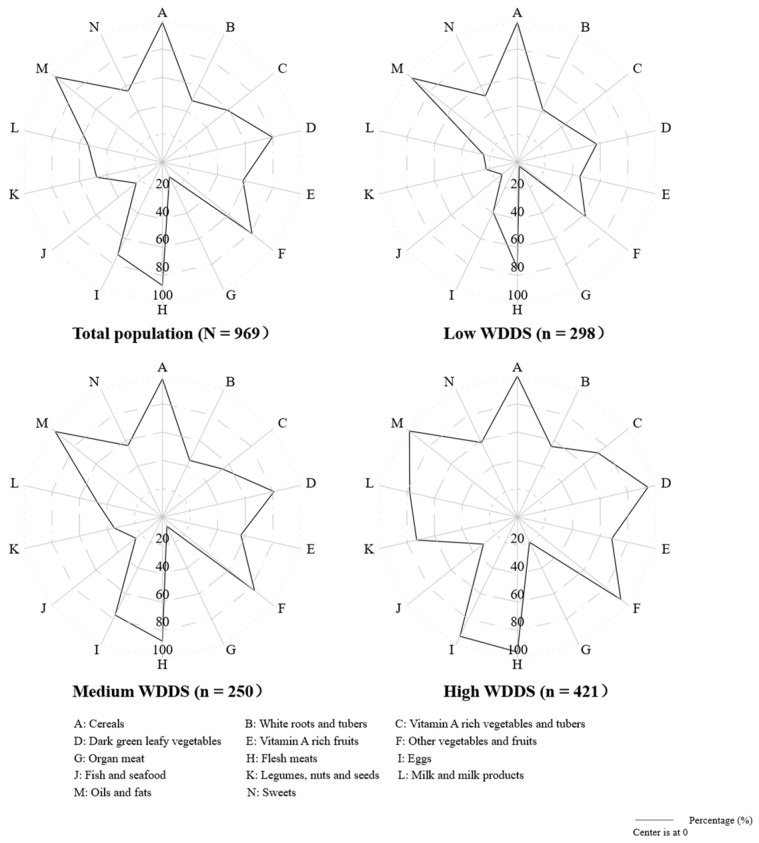
Radar charts for percentages (%) of the intake frequency of 14 food groups among pregnant women in rural Sichuan Province in China (N = 969).

The mean Women’s Dietary Diversity Score (WDDS) of the participants was 6.2 out of 9, with an SD of 1.39. Figure 2 provides a visual representation of the food intake, by food group, of the participants according to their WDDS. In addition, the results in Table A1 present comparisons of single food consumptions by WDDS terciles. According to the data, participants with high WDDSs consistently had the highest consumption of meat and fish (97.9%), dark green leafy vegetables (94.5%), other vitamin A-rich fruits and vegetables (96.9%), eggs (93.3%), other vegetables and fruits (93.3%), milk and milk products (77.9%), legumes, nuts, and seeds (72.9%), and organ meat (19.7%) (*p* < 0.05). In comparison, participants with low WDDSs had very low consumption rates of organ meat (3%), eggs (39.3%), milk and milk products (24.5%), and legumes, nuts, and seeds (22.5%).

### 3.4. Associations of Sociodemographic and Maternal Factors and Women’s Dietary Diversity Score with Measured Anaemia Status

The results in Table 2 show the associations between the sociodemographic and maternal factors of the participants and their (measured) anaemia status. According to these results, participants who were in their third trimester of pregnancy, were multiparous, had a lower education level, and had not taken folic acid supplements during pregnancy were more likely to have a higher severity of anaemia (*p* < 0.05). Moreover, participants with more severe anaemia were more likely to have low WDDSs (*p* < 0.05).

### 3.5. Multivariate Analyses for the Associations between Women’s Dietary Diversity Score, Anaemia Status, and Hb Concentrations

**Figure 3:**
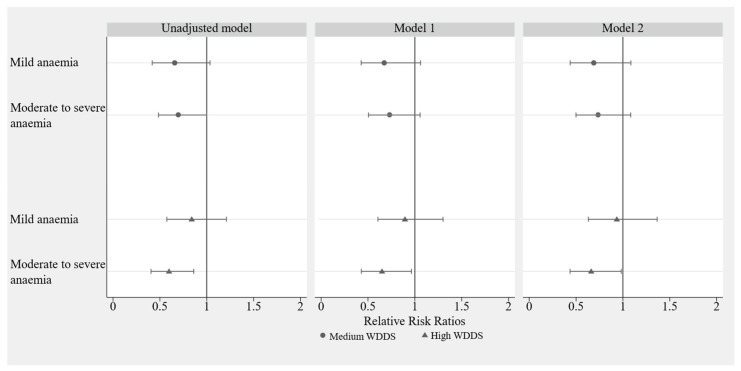
Associations between WDDS group and anaemia status among pregnant women in rural Sichuan Province in China (N = 969). Notes: Model 1 is adjusted for survey year, maternal age, occupation, education, SES, family size, gestational age, and parity. Model 2 is further adjusted for iron supplement intake and folic acid supplement intake during pregnancy.

Figure 3 illustrates the associations between WDDS terciles and anaemia status. The results of the unadjusted model demonstrate that participants with a high WDDS were less likely to be moderately or severely anaemic than participants with a low WDDS (RRR  =  0.59, 95% CI [0.40, 0.86], *p* < 0.01). After adjusting for survey year, maternal age, occupation, education, SES, family size, gestational age, and parity, associations were significant in Model 1 (RRR = 0.64, 95% CI [0.43, 0.96], *p* < 0.05); after further adjusting for iron supplement intake and folic acid supplement intake during pregnancy, associations remained significant in Model 2 (RRR =  0.65, 95% CI [0.44, 0.98], *p* < 0.05). In all models, there were no significant associations found for participants who were moderately to severely anaemic and had a medium WDDS. There were also no associations found between any of the explanatory variables and participants with mild anaemia (*p* > 0.05).

Table 3 presents the results of a subgroup analysis exploring the associations between WDDS terciles and anaemia status. The results show that there was a significant interaction between maternal age, parity, and WDDS terciles for participants with moderate to severe anaemia compared to participants with no anaemia (*p* for interaction < 0.05). This interaction was not observed for participants with mild anaemia. Additionally, participants under 25 years of age (the youngest age group in the sample) with a high or medium WDDS were less likely to have moderate to severe anaemia than participants under 25 years of age with a low WDDS. No such association was found in the older participants. Finally, participants with a medium WDDS who were primiparous (i.e., this was their first pregnancy) were found to be less likely to have moderate to severe anaemia compared to participants with a low WDDS who were also primiparous. No such associations were found among participants who were multiparous.

**Figure A1:**
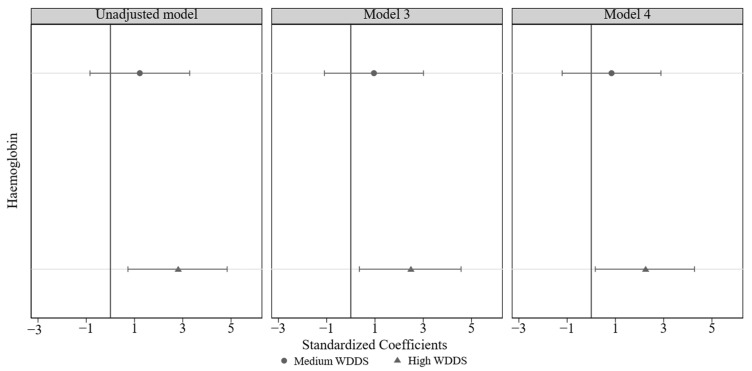
Associations between WDDS and haemoglobin levels of pregnant women in rural Sichuan Province in China (N = 969). Notes: Model 3 is adjusted for survey year, maternal age, occupation, education, SES, family size, gestational age, and parity. Model 4 is further adjusted for iron supplement intake and folic acid supplement intake during pregnancy.

We also conducted analysis exploring the associations between WDDS terciles and Hb concentrations. Because this analysis showed similar results to the previous analysis exploring associations between WDDS terciles and anaemia status, these results may be found in Figure A1 and Table A2.

## 4. Discussion

There is a robust body of literature documenting the importance of dietary diversity during pregnancy and the negative consequences of a less diverse diet [15,30]. In this cross-sectional survey conducted in rural areas of western China, the goal was to examine the relationship between the dietary diversity of women in their second or third trimester of pregnancy and their anaemia status. From a sample of 969 pregnant women, the results of this study demonstrate that the prevalence of anaemia was high among rural pregnant women, as over half of the women sampled had anaemia of some degree. Additionally, participants in the highest WDDS tercile had lower odds of being moderately to severely anaemic after adjusting for potential confounders. Finally, we observed a significant interaction between dietary diversity, age, and parity, as the inverse association between WDDS terciles and moderate to severe anaemia was mainly seen among participants who were younger than 25 and who were primiparous.

### 4.1. Prenatal Anaemia Prevalence

Perhaps one of the most important findings of this study is that anaemia remains a serious health problem among pregnant women living in rural areas of western China. According to WHO classifications, the prevalence of measured prenatal anaemia in our study population (54.3%) represents a severe public health problem [31]. In fact, the prevalence is similar to rates of anaemia published in 2019 for pregnant women living in low-income countries [32], such as Gambia (55.1%) and Niger (54.9%), and is higher than China’s latest national prevalence rate of 41.98% obtained from the Maternal and Newborn Health Monitoring System (2014–2018) [7]. Considering the socio-economic disparities, we believe that this study’s high prenatal anaemia prevalence needs to be paid more attention by specific governmental departments, and appropriate interventions are warranted.

### 4.2. Measured Versus Self-Reported Anaemia Status

This study’s results also reveal a significant discrepancy between self-reported and measured anaemia status, which is noteworthy. Specifically, we found that 10.6% of participants who reported not being anaemic were then measured to have moderate to severe anaemia, while 13.6% had mild anaemia. This lack of agreement could be attributed to rural pregnant women’s limited understanding of anaemia as a critical health issue during pregnancy, as well as their limited access to quality prenatal care in China’s rural regions [33]. In other words, if a pregnant woman is not aware of the health risks associated with pregnancy, this can affect her ability to make informed decisions and may lead to a reduced demand for prenatal care. This is particularly concerning for the population in this study, as access to prenatal care is already limited. The findings in this study are consistent with the suggestion that new public health efforts should be aimed at reducing the prevalence of prenatal anaemia in rural areas, and to accomplish this, policymakers should prioritize increasing awareness and knowledge of anaemia status during pregnancy, as well as improving the quality of prenatal care.

### 4.3. Dietary Intake

Regarding the consumption of different food groups, imbalanced dietary intake was detected among our sample. Most of the participants consumed animal-based foods (such as flesh meat and eggs) and dark green leafy vegetables. Nonetheless, their intake of food types that are considered protective against anaemia, such as organ meat, fish and seafood, legumes, nuts, and seeds, vitamin A-rich vegetables and fruits, and milk and milk products, were relatively low. The findings in this study are in part consistent with findings from the National Nutrition and Health Survey in China indicating that maternal diets during pregnancy are dominated by plant-based foods, and that the consumption of all animal-based foods except flesh meat is far below the recommended daily intake, resulting in suboptimal dietary diversity and micronutrient deficiencies [34]. Our results are also similar to those found by Liu et al., who showed that insufficient intake of milk and milk products, legumes, nuts, and seeds, fish and seafood, and organ meat was common among pregnant women living in the poor rural areas of Sichuan Province [35]. We believe that the relatively high price and low availability of key food items (such as fruits, milk and milk products, and fish and seafood) could be a reason behind why these food items are consumed less by our study population, which is still relatively poor. Furthermore, pregnant women in China—especially those living in rural areas—are more likely to adhere to traditional food taboos, and avoid eating some foods [36].

The suboptimal diet quality observed in our study population also could be attributable to the limited knowledge of the sample participants regarding essential food sources (e.g., meat, and milk and milk products) for preventing dietary anaemia. Because flesh meat and organ meat are iron-rich foods, and organ meat and milk and milk products are rich in vitamin A, these nutrients play a crucial role in the production of red blood cells and haemoglobin. Therefore, increased consumption of these food items could reduce the likelihood of nutritional anaemia [12]. This association between low dietary quality and inadequate knowledge about anaemia-preventing foods has been found in several studies [37]. Considering that the majority of rural pregnant women in China have low educational attainment [17], rural women may lack knowledge on topics such as what causes nutritional anaemia, which food sources are considered iron-rich and help to prevent nutritional anaemia, and how to combine different food sources for optimal nutrition.

### 4.4. Associations between WDDS Tercile and Anaemia

The current study also revealed a significant inverse relationship between WDDS and anaemia, meaning that participants in the highest WDDS tercile had lower odds of being moderately to severely anaemic. In previous studies, findings on the relationship between dietary diversity and anaemia remained mixed. While our findings are partly consistent with studies conducted in Ethiopia [15] and Cameroon [38], both of which showed an inverse association between dietary diversity and anaemia, two other studies carried out in the rural areas of northern Ghana [39] and Pakistan [40] found no significant association between dietary diversity and haemoglobin concentration in pregnant women. These inconsistencies in the literature could be because multiple factors contribute to the development of prenatal anaemia, and the contribution of each factor varies depending on various sociodemographic and maternal characteristics. However, in our study, the relationship between dietary diversity and prenatal anaemia or haemoglobin levels remained significant even after adjusting for potential confounding factors. Hence, based on our findings, we believe that adequate dietary diversity is a protective factor against anaemia among pregnant women in poor rural areas of China.

Additionally, the following plausible reasons could contribute to the association between WDDS and anaemia in this study. Participants with higher WDDSs were often the participants who consumed an increased percentage of nutrient-dense foods such as protein-based food (i.e., eggs, milk and dairy products, and legumes, nuts, and seeds), vitamin A-rich food (i.e., vitamin A-rich fruits and vegetables and dark green leafy vegetables), and bioavailable-heme-iron-rich food (i.e., red meat and organ meat). As the literature has indicated, improved dietary diversity leads to a higher intake of micronutrients, especially bioavailable heme iron, vitamin A, and vitamin C. These nutrients play a crucial role in iron mobilization, haemoglobin synthesis, and increasing iron absorption [41], thereby reducing the likelihood of developing nutritional anaemia.

What is noteworthy about the aforementioned inverse association between WDDS and anaemia was that we mainly observed it among younger, primiparous participants. We believe this finding may be explained as follows. The increased prevalence of anaemia in primiparous pregnancies may largely be attributed to the higher likelihood of a primiparous woman being younger [42] and having lower pre-pregnancy haemoglobin levels. In fact, being of a young age is a well-established risk factor for prenatal anaemia, as younger women may be more likely to have inadequate nutrient stores prior to conception and may not have access to sufficient amounts of nutrient-rich foods during pregnancy. Young women also may be more likely to have unplanned pregnancies, which can further increase the risk of developing anaemia if they are not adequately nutritionally prepared for pregnancy [43]. Therefore, given that our findings demonstrate the positive impact of adequate dietary diversity on prenatal anaemia, it is likely that these effects could be amplified among individuals with a higher risk of developing anaemia during pregnancy.

Finally, a fairly unique result from this study was that the correlation between WDDS and anaemia was identified only in pregnant women with moderate to severe anaemia, and not in those with mild anaemia. This finding aligns with the results of a study conducted in Ghana [44], which showed that significant associations between dietary diversity and anaemia were observed for moderate and severe anaemia but not mild anaemia among pregnant women. However, a study in rural India found no significant associations between dietary diversity and moderate or severe anaemia among women of reproductive age but identified an association for those with mild anaemia [45]. This inconsistency in the research may perhaps be attributed to variations in study populations and exposure categorization. Further studies are required to investigate these associations—or lack thereof—more comprehensively.

### 4.5. Limitations

The present study has several limitations that need to be taken into consideration. First, as the study’s cross-sectional design prevented us from making direct causal inferences, a causal association remains to be identified. Second, as the dietary data were all self-reported, participants might have overestimated their intake of some foods and underestimated their intake of other foods out of social desirability bias (i.e., the tendency of people to respond to survey questions or other forms of research in ways that they believe is socially acceptable or desirable). Third, although it has been recognized that nutrition during early pregnancy is associated with pregnancy outcomes, we were only able to investigate pregnant women in their second and third trimesters, as our study population had been chosen from lists provided by local health workers and most rural women in our study areas start prenatal care visits from their second trimester onwards. Finally, as our study participants lived in the rural western areas of Sichuan Province, the study results may not be representative of all rural areas in China. Further national representative studies are still warranted.

## 5. Conclusions

Our study found that the prevalence of prenatal anaemia in women living in rural areas of China is still quite high, and a more diverse diet is associated with a reduced risk of developing moderate to severe anaemia. Due to the high probability of pregnant women participants being unaware of their anaemia status, it is necessary to improve rural prenatal care to ensure better mother and child outcomes. Finally, as this study confirms that the adoption of a diverse diet may be a cost-effective intervention for lowering prenatal anaemia rates in rural China, enhancing education initiatives targeting young women or first-time mothers could aid in addressing these concerns.

## Figures and Tables

**Table 1:** Sociodemographic and maternal characteristics of pregnant women in rural Sichuan Province in China (N = 969).

Variables	N	Percent
Sociodemographic Characteristics		
Age		
16–24	243	25.1
25–29	391	40.4
30–45	335	34.6
Education level		
Primary school or below	142	14.7
Junior high school	435	44.9
Senior high school	232	23.9
College and above	160	16.5
Has a primary occupation		
No	767	79.2
Yes	202	20.8
SES index		
Low	304	31.4
Medium	292	30.1
High	373	38.5
Family size		
≤4	517	53.3
≥5	452	46.7
Maternal Characteristics		
Gestational stage		
Second trimester	425	43.9
Third trimester	544	56.1
Parity		
Primigravida	331	34.2
Multigravida	638	65.8
Iron supplements taken during pregnancy		
No	586	60.5
Yes	383	39.5
Folic acid supplements taken during pregnancy		
No	106	10.9
Yes	863	89.1

**Table 2:** Associations between sociodemographic and maternal factors and anaemia status in rural Sichuan Province in China (N = 969).

Variables	No Anaemia (*n* = 443)	Mild Anaemia (*n* = 277)	Moderate to Severe Anaemia (*n* = 249)	*p* Value
Sociodemographic Characteristics				
Age				
16–24	111 (25.1)	67 (24.2)	65 (26.1)	0.099
25–29	176 (39.7)	128 (46.2)	87 (34.9)	
30–45	156 (35.2)	82 (29.6)	97 (39.0)	
Education level				
Primary school or below	57 (12.9)	32 (11.6)	53 (21.3)	0.025
Junior high school	193 (43.6)	134 (48.4)	108 (43.4)	
Senior high school	113 (25.5)	64 (23.1)	55 (22.1)	
College and above	80 (18.1)	47 (17.0)	33 (13.3)	
Has a primary occupation				
No	336 (75.8)	225 (81.2)	206 (82.7)	0.061
Yes	107 (24.2)	52 (18.8)	43 (17.3)	
SES index				
Low	136 (30.7)	83 (30.0)	85 (34.1)	0.720
Medium	129 (29.1)	88 (31.8)	75 (30.1)	
High	178 (40.2)	106 (38.3)	89 (35.7)	
Maternal Characteristics				
Gestational age				
Second trimester	210 (47.4)	122 (44.0)	93 (37.3)	0.038
Third trimester	233 (52.6)	155 (56.0)	156 (62.7)	
Parity				
Primigravida	173 (39.1)	87 (31.4)	71 (28.5)	0.010
Multigravida	270 (60.9)	190 (68.6)	178 (71.5)	
Iron supplements taken during pregnancy				
Yes	181 (40.9)	108 (39.0)	94 (37.8)	0.71
No	262 (59.1)	169 (61.0)	155 (62.2)	
Folic acid supplements taken during pregnancy				
Yes	31 (7.0)	32 (11.6)	43 (17.3)	<0.001
No	412 (93.0)	245 (88.4)	206 (82.7)	
WDDS				
By mean (SD)	6.3 (1.4)	6.2 (1.4)	6.0 (1.4)	0.014
By terciles				
Low	118 (26.6)	89 (32.1)	91 (36.5)	0.029
Medium	123 (27.8)	61 (22.0)	66 (26.5)	
High	202 (45.6)	127 (45.8)	92 (36.9)	

**Table 3:** Subgroup analysis of the associations between WDDS groups and anaemia status among pregnant women in rural Sichuan Province in China (N = 969).

Variables	Mild Anaemia				Moderate–Severe Anaemia			
	WDDS (Low)	WDDS (Medium)	WDDS (High)	*p* for Interaction	WDDS (Low)	WDDS (Medium)	WDDS (High)	*p* for Interaction
Age								
<25	1 (reference)	0.84 (0.34, 2.08)	0.75 (0.34, 1.69)	0.854	1 (reference)	0.39 (0.16, 0.91)	0.30 (0.14, 0.70)	0.022
(25–29)	1 (reference)	0.72 (0.36, 1.45)	1.19 (0.63, 2.27)		1 (reference)	0.96 (0.49, 1.89)	0.83 (0.37, 1.84)	
≥30	1 (reference)	0.53 (0.25, 1.16)	0.87 (0.45, 1.71)		1 (reference)	1.05 (0.50, 2.19)	1.01 (0.51, 2.00)	
Parity								
Primigravida	1 (reference)	0.81 (0.36, 1.83)	0.77 (0.38, 1.54)	0.295	1 (reference)	0.46 (0.21, 1.01)	0.37 (0.19, 0.70)	0.012
Multigravida	1 (reference)	0.61 (0.32, 1.14)	1.19 (0.73, 1.95)		1 (reference)	0.76 (0.46–1.26)	0.89 (0.49–1.61)	
Gestational age								
Second trimester	1 (reference)	0.55 (0.28, 1.08)	0.93 (0.51, 1.68)	0.956	1 (reference)	0.54 (0.30, 0.99)	0.55 (0.30, 0.98)	0.573
Third trimester	1 (reference)	0.78 (0.43, 1.41)	0.97 (0.50, 1.69)		1 (reference)	0.93 (0.56, 1.57)	0.76 (0.44, 1.31)	
Iron supplements taken during pregnancy								
No	1 (reference)	0.51 (0.27, 0.96)	0.92 (0.50, 1.69)	0.867	1 (reference)	0.47 (0.26, 0.85)	0.52 (0.27, 0.99)	0.248
Yes	1 (reference)	0.84 (0.46, 1.54)	0.96 (0.59, 1.56)		1 (reference)	1.00 (0.59, 1.70)	0.79 (0.47, 1.34)	
Folic acid supplements taken during pregnancy								
No	1 (reference)	1.91 (0.50, 7.29)	1.27 (0.34, 4.71)	0.943	1 (reference)	0.91 (0.21, 3.97)	1.33 (0.37, 4.72)	0.744
Yes	1 (reference)	0.68 (0.42, 1.08)	0.91 (0.61, 1.36)		1 (reference)	0.75 (0.48, 1.17)	0.65 (0.41, 1.04)	

## Data Availability

The data presented in this study are available upon request from the corresponding author. The data are not publicly available.

## Appendix A

**Table A1:** Comparisons of participants’ food consumption in a 24 h period among low-, medium-, and high-WDDS groups in rural Sichuan Province in China (N = 969).

Consumption by Food Group (Not Consumed = No, Consumed = Yes)	Low-WDDS Group (%) (*n* = 298)	Medium-WDDS Group (%) (*n* = 250)	High-WDDS Group (%) (*n* = 421)	*p* Value
Starchy staples				
No	3 (1.0)	3 (1.2)	2 (0.5)	
Yes	295 (99.0)	247 (98.8)	419 (99.5)	0.55
Dark green leafy vegetables				
No	127 (42.6)	48 (19.2)	23 (5.5)	
Yes	171 (57.4)	202 (80.8)	398 (94.5)	<0.001
Other vitamin A-rich fruits and vegetables				
No	106 (35.6)	55 (22.0)	13 (3.1)	
Yes	192 (64.4)	195 (78.0)	408 (96.9)	<0.001
Other fruit and vegetables				
No	115 (38.6)	42 (16.8)	28 (6.7)	
Yes	183 (61.4)	208 (83.2)	393 (93.3)	<0.001
Organ meat				
No	289 (97.0)	232 (92.8)	338 (80.3)	
Yes	9 (3.0)	18 (7.2)	83 (19.7)	<0.001
Meat and fish				
No	66 (22.1)	17 (6.8)	9 (2.1)	
Yes	232 (77.9)	233 (93.2)	412 (97.9)	<0.001
Eggs				
No	181 (60.7)	58 (23.2)	28 (6.7)	
Yes	117 (39.3)	192 (76.8)	393 (93.3)	<0.001
Legumes, nuts, and seeds				
No	231 (77.5)	163 (65.2)	114 (27.1)	
Yes	67 (22.5)	87 (34.8)	307 (72.9)	<0.001
Milk and milk products				
No	225 (75.5)	132 (52.8)	93 (22.1)	
Yes	73 (24.5)	118 (47.2)	328 (77.9)	<0.001

**Table A2:** Subgroup analysis of the associations between WDDS and haemoglobin levels among pregnant women in rural Sichuan Province in China (N = 969).

Variables	Haemoglobin			
	WDDS (Low)	WDDS (Medium)	WDDS (High)	*p* for Interaction
Age				
<25	1 (reference)	4.03 [−0.36, 8.43]	6.67 [2.12, 11.23]	0.126
[25–29]	1 (reference)	−0.60 [−3.81, 2.61]	0.87 [−2.60, 4.34]	
≥30	1 (reference)	−0.63 [−4.77, 3.52]	0.19 [−3.32, 3.70]	
Parity				
Primigravida	1 (reference)	3.75 [0.03, 7.48]	5.24 [1.50, 8.98]	0.029
Multigravida	1 (reference)	1.02 [−1.64, 3.66]	0.64 [−2.07, 3.35]	
Gestational age				
Second trimester	1 (reference)	2.19 [−0.64, 5.03]	2.65 [−0.31, 5.61]	0.559
Third trimester	1 (reference)	−0.29 [−3.20, 2.61]	1.66 [−1.24, 4.57]	
Iron supplements taken during pregnancy				
No	1 (reference)	3.29 [0.35, 6.22]	3.76 [0.74, 6.78]	0.151
Yes	1 (reference)	−1.01 [−3.71, 1.68]	0.88 [−1.52, 3.28]	
Folic acid supplements taken during pregnancy				
No	1 (reference)	1.84 [−5.41, 9.09]	2.15 [−3.45, 7.76]	0.844
Yes	1 (reference)	0.65 [−3.45, 7.76]	2.16 [−0.20, 4.51]	

## References

[1] Balarajan Y., Ramakrishnan U., Özaltin E., Shankar A.H., Subramanian S. (2011). Anaemia in Low-Income and Middle-Income Countries. Lancet.

[2] World Health Organization Anaemia. https://www.who.int/health-topics/anaemia.

[3] Stevens G.A., Paciorek C.J., Flores-Urrutia M.C., Borghi E., Namaste S., Wirth J.P., Suchdev P.S., Ezzati M., Rohner F., Flaxman S.R. (2022). National, Regional, and Global Estimates of Anaemia by Severity in Women and Children for 2000-19: A Pooled Analysis of Population-Representative Data. Lancet Glob. Health.

[4] Haider B.A., Olofin I., Wang M., Spiegelman D., Ezzati M., Fawzi W.W. (2013). Nutrition Impact Model Study Group (Anaemia). Anaemia, Prenatal Iron Use, and Risk of Adverse Pregnancy Outcomes: Systematic Review and Meta-Analysis. BMJ.

[5] Iqbal S., Ekmekcioglu C. (2019). Maternal and Neonatal Outcomes Related to Iron Supplementation or Iron Status: A Summary of Meta-Analyses. J. Matern. Fetal Neonatal Med..

[6] Qiao J., Wang Y., Li X., Jiang F., Zhang Y., Ma J., Song Y., Ma J., Fu W., Pang R. (2021). A Lancet Commission on 70 Years of Women’s Reproductive, Maternal, Newborn, Child, and Adolescent Health in China. Lancet.

[7] Hu H., Huang A., Yang Q., Zhao W., Ma Y., Di J. (2020). Prevalence and Risk Factors of Anemia of Pregnant Women—6 Provinces in China, 2014–2018. China CDC Wkly..

[8] Zhao S.Y., Jing W.Z., Liu J., Liu M. (2018). Prevalence of anemia during pregnancy in China, 2012–2016: A Meta-analysis. Zhonghua Yu Fang Yi Xue Za Zhi.

[9] The General Office of the State Council National Nutrition Plan (2017–2030). http://www.gov.cn/xinwen/2017-07/13/content_5210199.htm.

[10] The Central People’s Government of the People’s Republic of China Healthy China Action Plan (2019–2030). http://www.gov.cn/xinwen/2019-07/15/content_5409694.htm.

[11] Lopez A., Cacoub P., Macdougall I.C., Peyrin-Biroulet L. (2016). Iron Deficiency Anaemia. Lancet.

[12] World Health Organization Nutritional Anaemias: Tools for Effective Prevention and Control. https://www.who.int/publications-detail-redirect/9789241513067.

[13] Brazier A.K.M., Lowe N.M., Zaman M., Shahzad B., Ohly H., McArdle H.J., Ullah U., Broadley M.R., Bailey E.H., Young S.D. (2020). Micronutrient Status and Dietary Diversity of Women of Reproductive Age in Rural Pakistan. Nutrients.

[14] Lee S.E., Talegawkar S.A., Merialdi M., Caulfield L.E. (2013). Dietary Intakes of Women during Pregnancy in Low- and Middle-Income Countries. Public Health Nutr..

[15] Zerfu T.A., Umeta M., Baye K. (2016). Dietary Diversity during Pregnancy Is Associated with Reduced Risk of Maternal Anemia, Preterm Delivery, and Low Birth Weight in a Prospective Cohort Study in Rural Ethiopia. Am. J. Clin. Nutr..

[16] Cheng Y., Dibley M.J., Zhang X., Zeng L., Yan H. (2009). Assessment of Dietary Intake among Pregnant Women in a Rural Area of Western China. BMC Public Health.

[17] Ma Y., Gao Y., Li J., Sun A., Wang B., Zhang J., Dill S.-E., Medina A., Rozelle S. (2020). Maternal Health Behaviors during Pregnancy in Rural Northwestern China. BMC Pregnancy Childbirth.

[18] Kangalgil M., Sahinler A., Kırkbir I.B., Ozcelik A.O. (2021). Associations of Maternal Characteristics and Dietary Factors with Anemia and Iron-Deficiency in Pregnancy. J. Gynecol. Obstet. Hum. Reprod..

[19] Rezk M., Marawan H., Dawood R., Masood A., Abo-Elnasr M. (2015). Prevalence and Risk Factors of Iron-Deficiency Anaemia among Pregnant Women in Rural Districts of Menoufia Governorate, Egypt. J. Obstet. Gynaecol..

[20] Nasir B.B., Fentie A.M., Adisu M.K. (2020). Adherence to Iron and Folic Acid Supplementation and Prevalence of Anemia among Pregnant Women Attending Antenatal Care Clinic at Tikur Anbessa Specialized Hospital, Ethiopia. PLoS ONE.

[21] Food and Agriculture Organization of the United Nations Guidelines for Measuring Household and Individual Dietary Diversity. https://www.fao.org/agrifood-economics/publications/detail/en/c/122321/.

[22] Hoddinott J., Yohannes Y. (2002). Dietary Diversity as a Food Security Indicator.

[23] Arimond M., Wiesmann D., Becquey E., Carriquiry A., Daniels M.C., Deitchler M., Fanou-Fogny N., Joseph M.L., Kennedy G., Martin-Prevel Y. (2010). Simple Food Group Diversity Indicators Predict Micronutrient Adequacy of Women’s Diets in 5 Diverse, Resource-Poor Settings. J. Nutr..

[24] Wang S., Liu A., Guo W. (2021). Public and Commercial Medical Insurance Enrollment Rates of Rural-to-Urban Migrants in China. Front. Public Health.

[25] Savy M., Martin-Prével Y., Sawadogo P., Kameli Y., Delpeuch F. (2005). Use of Variety/Diversity Scores for Diet Quality Measurement: Relation with Nutritional Status of Women in a Rural Area in Burkina Faso. Eur. J. Clin. Nutr..

[26] Alamirew S.K., Lemke S., Stadlmayr B., Freyer B. (2023). Dietary Behaviour and Sociocultural Determinants of Dietary Diversity among Rural Women of Reproductive Age: A Case of Amhara Region, Ethiopia. Nutrients.

[27] Xu H., Du S., Liu A., Zhang Q., Ma G. (2022). Low Dietary Diversity for Recommended Food Groups Increases the Risk of Obesity among Children: Evidence from a Chinese Longitudinal Study. Nutrients.

[28] Vyas S., Kumaranayake L. (2006). Constructing Socio-Economic Status Indices: How to Use Principal Components Analysis. Health Policy Plan..

[29] von Schenck H., Falkensson M., Lundberg B. (1986). Evaluation of “HemoCue”, a New Device for Determining Hemoglobin. Clin. Chem..

[30] Kheirouri S., Alizadeh M. (2021). Maternal Dietary Diversity during Pregnancy and Risk of Low Birth Weight in Newborns: A Systematic Review. Public Health Nutr..

[31] World Health Organization (2008). World Health Organization Worldwide Prevalence of Anaemia 1993–2005: WHO Global Database on Anaemia.

[32] World Health Organization Prevalence of Anaemia in Pregnant Women (Aged 15–49) (%). https://www.who.int/data/gho/data/indicators/indicator-details/GHO/prevalence-of-anaemia-in-pregnant-women-(-).

[33] Gao Y., Zhou H., Singh N.S., Powell-Jackson T., Nash S., Yang M., Guo S., Fang H., Alvarez M.M., Liu X. (2017). Progress and Challenges in Maternal Health in Western China: A Countdown to 2015 National Case Study. Lancet Glob. Health.

[34] Chang J.L., Wang Y. (2016). Reports of China Nutrition and Health Survey (2010–2013).

[35] Liu Y., Feng X., Luo B. (2016). Investigation on the dietary behaviors of pregnant women in rural areas of poverty-stricken counties in Sichuan Province. Matern. Child Health Care China.

[36] Yang C., Zhao A., Lan H., Ren Z., Zhang J., Szeto I.M.-Y., Wang P., Zhang Y. (2021). Association between Dietary Quality and Postpartum Depression in Lactating Women: A Cross-Sectional Survey in Urban China. Front. Nutr..

[37] Yamashita T., Roces R.E.D., Ladines-Llave C., Tuliao M.T.R., Kamau M.W., Yamada C., Tanaka Y., Shimazawa K., Iwamoto S., Matsuo H. (2021). Dietary Intake Quality Is Affected by Knowledge and Dietary Intake Frequency among Pregnant Women in Muntinlupa, Philippines: A Cross-Sectional Study. Int. J. Environ. Res. Public Health.

[38] Jugha V.T., Anchang-Kimbi J.K., Anchang J.A., Mbeng K.A., Kimbi H.K. (2021). Dietary Diversity and Its Contribution in the Etiology of Maternal Anemia in Conflict Hit Mount Cameroon Area: A Cross-Sectional Study. Front. Nutr..

[39] Saaka M., Oladele J., Larbi A., Hoeschle-Zeledon I. (2017). Dietary Diversity Is Not Associated with Haematological Status of Pregnant Women Resident in Rural Areas of Northern Ghana. J. Nutr. Metab..

[40] Ali F., Thaver I., Khan S.A. (2014). Assessment of Dietary Diversity and Nutritional Status of Pregnant Women in Islamabad, Pakistan. J. Ayub Med. Coll. Abbottabad.

[41] Péneau S., Dauchet L., Vergnaud A.-C., Estaquio C., Kesse-Guyot E., Bertrais S., Latino-Martel P., Hercberg S., Galan P. (2008). Relationship between Iron Status and Dietary Fruit and Vegetables Based on Their Vitamin C and Fiber Content. Am. J. Clin. Nutr..

[42] Delpisheh A., Attia E., Drammond S., Brabin B.J. (2006). Adolescent Smoking in Pregnancy and Birth Outcomes. Eur. J. Public Health.

[43] Wu Y., Ye H., Liu J., Ma Q., Yuan Y., Pang Q., Liu J., Kong C., Liu M. (2020). Prevalence of Anemia and Sociodemographic Characteristics among Pregnant and Non-Pregnant Women in Southwest China: A Longitudinal Observational Study. BMC Pregnancy Childbirth.

[44] Agbozo F., Abubakari A., Der J., Jahn A. (2020). Maternal Dietary Intakes, Red Blood Cell Indices and Risk for Anemia in the First, Second and Third Trimesters of Pregnancy and at Predelivery. Nutrients.

[45] Jin Y., Talegawkar S.A., Sedlander E., DiPietro L., Parida M., Ganjoo R., Aluc A., Rimal R. (2022). Dietary Diversity and Its Associations with Anemia among Women of Reproductive Age in Rural Odisha, India. Ecol. Food Nutr..

